# Preoperative low skeletal muscle mass as a risk factor for pharyngocutaneous fistula and decreased overall survival in patients undergoing total laryngectomy

**DOI:** 10.1002/hed.25638

**Published:** 2019-01-20

**Authors:** Sandra I. Bril, Thomas F. Pezier, Bernard M. Tijink, Luuk M. Janssen, Weibel W. Braunius, Remco de Bree

**Affiliations:** ^1^ Department of Head and Neck Surgical Oncology University Medical Center Utrecht Utrecht The Netherlands; ^2^ Department of Head and Neck Surgery Netherlands Cancer Institute Amsterdam The Netherlands

**Keywords:** body composition, computer‐assisted image analysis, head and neck neoplasms, postoperative complications, sarcopenia, skeletal muscle mass, surgery, survival

## Abstract

**Background:**

Low skeletal muscle mass (SMM) is associated with postoperative complications, prolonged hospital stay, and short overall survival (OS) in surgical oncology. We aimed to investigate this association in patients undergoing total laryngectomy (TL).

**Methods:**

A retrospective study was performed of patients undergoing TL. SMM was measured using CT or MRI scans at the level of the third cervical vertebra (C3).

**Results:**

In all, 235 patients were included. Low SMM was observed in 109 patients (46.4%). Patients with low SMM had more pharyngocutaneous fistulas (PCFs) than patients with normal SMM (34.9% vs 20.6%; *P* = .02) and prolonged hospital stay (median, 17 vs 14 days; *P* < .001). In multivariate analysis, low SMM (hazards ratio, 1.849; 95% confidence interval, 1.202‐2.843) and high N stage were significant prognosticators of decreased OS.

**Conclusion:**

Low SMM is associated with PCF and prolonged hospital stay in patients undergoing TL. Low SMM is an independent prognostic factor for shorter OS.

**ABBREVIATIONS**
AJCC: American Joint Committee on Cancer
ASA: American Society of Anesthesiologist
C3: third cervical vertebra
CI: confidence interval
CSA: cross‐sectional muscle area
DSS: disease‐specific survival
HNC: head and neck cancer
HU: Hounsfield units
L3: third lumbar vertebra
OR: odds ratio
OS: overall survival
PCF: pharyngocutaneous fistula
SM: skeletal muscle
SMI: skeletal muscle index
SMM: skeletal muscle mass
TL: total laryngectomy

## 1 INTRODUCTION

Total laryngectomy (TL) with or without (partial) pharyngectomy, often followed by postoperative (chemo)radiotherapy is a curative treatment option for patients with advanced stage primary laryngeal or hypopharyngeal cancer. TL can also be used to salvage patients with recurrent disease after failure of initial organ preserving treatment with (chemo)radiotherapy or in patients without (current) cancer but with a dysfunctional larynx.^1^, ^2^ TL is an invasive surgical procedure and is associated with significant morbidity and mortality, as well as a reduced quality of life after surgery.^3^

Postoperative complications including the occurrence of a pharyngocutaneous fistula (PCF) are common and difficult‐to‐treat problems after TL.^1^, ^2^, ^3^, ^4^ Up to 30% of patients develop PCF, which may require additional surgery, prolongs feeding tube dependency, and increases hospital stay.^5^ Previously described risk factors for the occurrence of a PCF include prior chemoradiotherapy with platinum‐based chemotherapy, hypopharyngeal cancer, extensive pharyngeal resection and reconstruction, additional neck dissection, and low BMI. The occurrence of a PCF may also cause delay of postoperative (chemo)radiotherapy, thus jeopardizing optimal oncological treatment.^6^, ^7^

The radiological assessment of body composition has increasingly gained attention in oncological research over the last decade.^8^, ^9^ Specifically a low skeletal muscle mass (SMM), also termed sarcopenia, has been related to negative outcomes in variety of tumor types and treatments. In geriatric patients, sarcopenia is defined as a geriatric syndrome characterized by the age‐related loss of muscle mass and/or muscle function or decreased physical status.^10^ In oncological patients, often only SMM assessed as muscle function is rarely measured during routine clinical practice.^11^

In oncological patients, SMM is most commonly assessed on abdominal CT imaging at the level of the third lumbar vertebra (L3).^12^, ^13^ Abdominal CT is routinely performed during diagnostic work‐up and follow‐up of many patients with cancer, and thus imaging is routinely available for analysis without any extra burden for the patient or health care‐related costs.

Low SMM has been related to more postoperative complications, prolonged hospital stay, and decreased survival in surgical oncology.^14^, ^15^ In head and neck cancer (HNC), the predictive and prognostic value of low SMM has not yet been researched as thoroughly. Abdominal CT imaging is not routinely performed in patients with HNC and is often only available in a preselected patient group with advanced disease and high risk features for distant metastasis. Recently, a novel SMM assessment method at the level of the third cervical vertebra (C3) was published.^16^ Imaging at the level of C3 is almost always available in patients with HNC, allowing for the routine assessment of SMM.

In this article, we aim to investigate whether preoperative low SMM, as measured using CT or MRI at the level of C3, is a significant predictor of postoperative complications including PCF, prolonged hospital stay, and decreased overall survival (OS) in a large consecutive cohort of patients undergoing TL for any indication.

## 2 PATIENTS AND METHODS

The design of this study was approved by the Medical Ethical Research Committee of the University Medical Center Utrecht (ID 17‐365/C). The research was conducted in accordance with the Declaration of Helsinki.

### 2.1 Patient and study design

A retrospective case note review was performed of all consecutive patients who had undergone TL between January 2008 and May 2017 at the University Medical Center, Utrecht, the Netherlands, a tertiary referral center for patients with HNC. All patients were discussed in the local multidisciplinary tumor board prior to and after surgery. Patients without recent CT or MRI scans (less than 3 months) of the head and neck area prior to TL were excluded. Patients who had severe dental artifacts at the level of C3 that impeded accurate assessment of SMM were also excluded.

Patients’ demographic, staging, treatment, and outcome data were collected using electronic patient records. Both versions 6 and 7 of the American Joint Committee on Cancer (AJCC) manual were used for staging as the study period straddled the change in 2009.^17^ All patients were discussed in local tumor board meetings and underwent TL with or without (partial) pharyngectomy and with or without additional lymph node dissection either as a primary treatment, a salvage treatment, or a functional treatment for a dysfunctional larynx.

Five dedicated head and neck surgical oncologists performed all total laryngectomies during the time period. Operating records were checked for details of the surgery, neck dissection, and primary pharyngeal closure or flap reconstruction of the pharynx. Prior treatment with radiotherapy or chemoradiotherapy for HNC was recorded. Postoperative adjuvant treatment was also recorded. The American Society of Anesthesiologist's physical status classification was recorded as a surrogate marker for comorbidities.^18^ Postoperative complications were graded according to the Clavien‐Dindo classification of Surgical Complications.^19^ Severe complications were defined as Clavien‐Dindo grade 3A or higher. Of specific interest was the occurrence of PCF which was scored separately to the other postoperative complications. The occurrence of PCF was defined as a clinical fistula requiring any form of conservative or surgical treatment. Duration of hospital stay was recorded as the time in days between the date of TL and date of first hospital discharge.

Follow‐up/survival data were retrieved up until August 31, 2017. OS was defined as the time elapsed between the date of TL and the date of death. Disease‐specific survival (DSS) was defined as any patient who had died as a result of the current HNC diagnosis or as a result of the surgical procedure. Survival status was checked in the patient medical records in our hospital. Patients are routinely contacted during the first 5 years of follow‐up after TL.

### 2.2 SMM measurement

**Figure 1:**
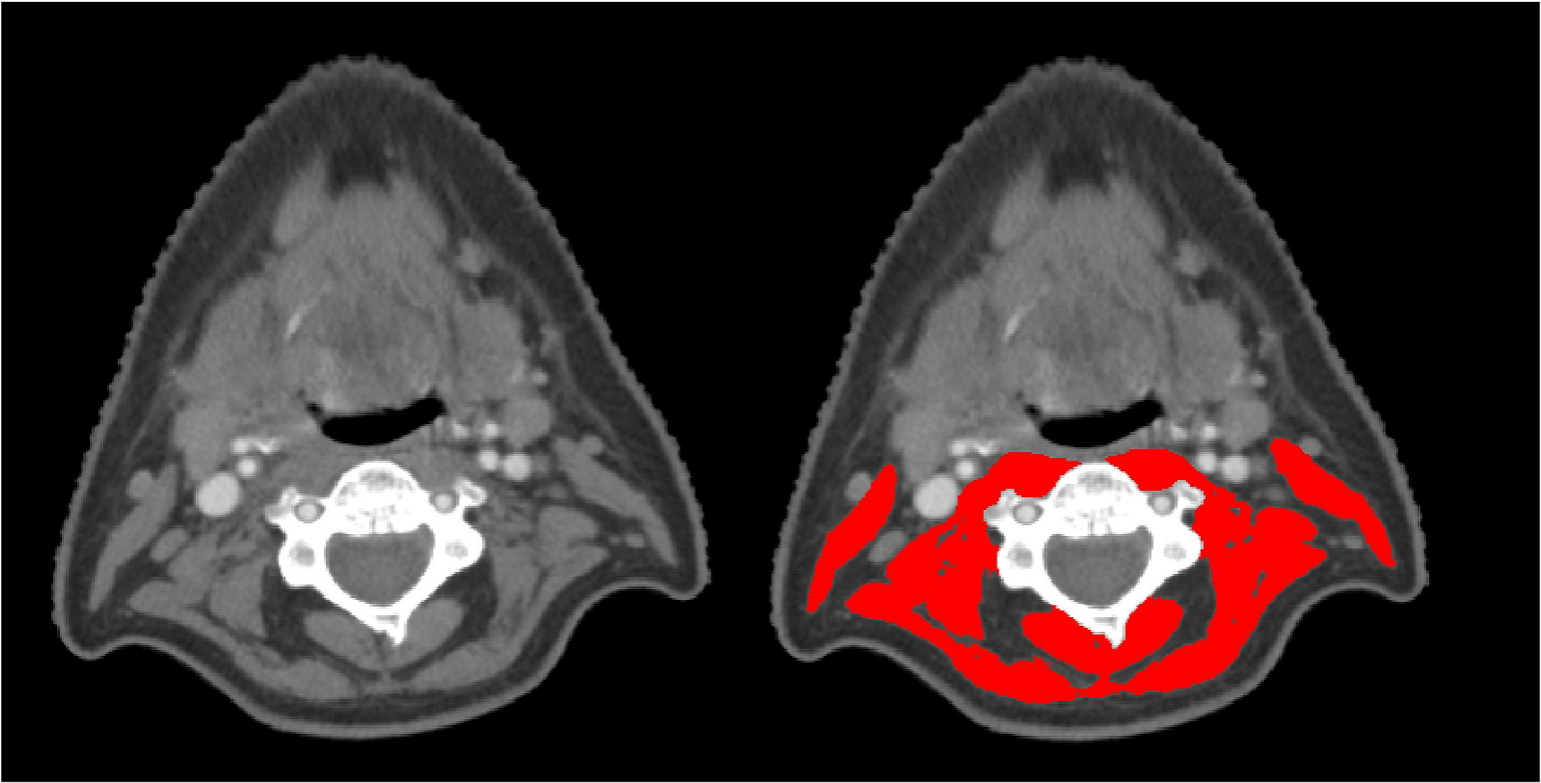
Example of segmentation of skeletal muscle tissue at the level of third cervical vertebra (C3). Two identical axial CT‐slides at the level of C3; the left CT slide shows the skeletal muscle tissue unsegmented, while the right image shows the paravertebral muscles and both sternocleidomastoid muscles segmented in red, using SliceOmatic software. Hounsfield unit (HU) ranges are set at −29 to +150 HU for optimal identification of skeletal muscle tissue [Color figure can be viewed at wileyonlinelibrary.com]

SMM was measured on pretreatment CT or MRI scans of the head and neck area at the level of C3 using a previously published method.^16^ Whenever possible, CT imaging was used instead of MRI. In brief, the first slide at the level of C3 when scrolling from caudal to cranial direction to show both transverse processes and the entire vertebral arc was selected for segmentation of skeletal muscle (SM) tissue. For CT imaging, SM area was defined as the pixel area within a radiodensity between −29 and +150 Hounsfield units (HU), which is specific for SM tissue.^20^ For MRI, SM tissue was carefully segmented and any intramuscular fatty tissue was manually excluded. Segmentation of SM tissue was manually performed by a single researcher (S.B.) using the commercially available software package SliceOmatic (Tomovision, Magog, Quebec, Canada). An example of SM tissue segmentation at the level of C3 is shown in Figure 1. After a learning period, the measurement of SMM requires 5 to 10 minutes per CT scan, and up to 15 minutes per MRI scan.

From SM area at C3, SM area at the level of L3 was predicted using the previously published formula 1.^16^ The SM area at L3 was then normalized for height to calculate the lumbar skeletal muscle index (lumbar SMI), as shown in formula 2.^12^ Low SMM was defined as a lumbar SMI lower than 43.2 cm^2^/m^2^. This recently published cutoff value was established in a separate cohort of patients with HNC.^21^

Formula 1:

$$\mathrm{CSA}\;\mathrm{at}\;L3\;\left({\mathrm{cm}}^{2}\right)=27.304+1.363*\mathrm{CSA}\;\mathrm{at}\;C3\;\left({\mathrm{cm}}^{2}\right)-0.671*\mathrm{Age}\;\left(\text{years}\right)+0.640*\text{Weight}\;\left(\mathrm{kg}\right)+26.442*\mathrm{Sex}\;\left(\mathrm{Sex}=1\;\text{for female},2\;\text{for male}\right)$$

Formula 2:

$$\text{Lumbar}\;\mathrm{SMI}\;\left({\mathrm{cm}}^{2}/m^{2}\right)=\mathrm{CSA}\;\mathrm{at}\;L3/\text{length}\;\left(m^{2}\right)$$

### 2.3 Statistical analysis

Categorical data are represented as a number and percentage of the total. All statistical analyses were performed using the IBM SPSS Statistics version 21.0 software package (Chicago, Illinois). A test for normality (Kolmogorov‐Smirnoff test) and histograms were used to assess whether continuous variables were normally distributed. Continuous data are represented as mean ± SD if normally distributed, and median ± interquartile range if skewed. Fisher's exact tests, Pearson Chi square tests, independent sample *t* tests, and Mann‐Whitney *U* tests were used to assess group differences. Binary logistic regression analysis was used to assess the association between low SMM and the occurrence of PCF. Kaplan‐Meier estimates were used to visualize the relationship between low SMM and survival outcomes. Univariate and multivariate Cox proportional hazard models were used to assess the association between low SMM and OS. Parameters entered as covariates in regression analysis were chosen based on known or expected association with outcomes. All analyses were two‐sided and *P* ≤ .05 was considered significant.

## 3 RESULTS

Between January 2008 and June 2017, 245 patients underwent TL at our institution. Of these 245 patients, 235 (95.9%) had appropriate imaging available and were included in this study. Mean interval between imaging and TL was 3 weeks; maximum was 3 months. Four patients without recent imaging available that were excluded from this study were diagnosed with a dysfunctional larynx and underwent functional laryngectomy, two patients had severe dental artifacts on the imaging impeding accurate SMM assessment at the level of C3, and four patients had irretrievable scans.

### 3.1 Patient demographics

Patient, disease, and surgical characteristics are presented in Table 1 and the outcomes after surgery in Table 2. During the study period, 108 patients underwent primary TL, 114 patients underwent salvage TL, and 13 patients underwent a functional TL. A complication of any grade occurred in 64.3% of patients after TL. The most commonly noted complication was a Clavien‐Dindo grade I transient hypocalcaemia after surgery, necessitating short‐term calcium monitoring and supplementation. Severe complications occurred in 69 patients (29.4%). A PCF occurred in 64 patients (27.2%), which required surgical closure in 40 patients (17.0%). Mean duration of hospital stay after TL was 21 days. There were four postoperative deaths (1.7%). At the time of concluding this study, with a median time of 62.7 months after TL, 134 (57.0%) patients had died of any cause and 101 (43.0%) were alive.

**Table 1:** Patient, disease, and surgical characteristics

Characteristic	All patients (*n* = 235)	Patients with low SMM (*n* = 109)	Patients with normal SMM (*n* = 126)	*P* value
Sex				
Male	193 (82.1)	72 (66.1)	121 (96.0)	**<.001** a
Female	42 (17.9)	37 (33.9%)	5 (4.0)	
Age	64.7 (9.1)	65.6 (8.9)	63.8 (9.3)	.13^b^
BMI	23.9 (5.0)	21.1 (3.8)	26.4 (4.6)	**<.001** b
Smoking				
Never	6 (2.6)	4 (3.7)	2 (1.6)	**.04** c
Current	112 (47.6)	60 (55.0)	52 (41.3)	
Former	117 (49.8)	45 (41.3)	72 (57.1)	
Alcohol abuse				
No	159 (67.7)	72 (66.0)	87 (69.0)	.72^c^
Yes, current	26 (11.1)	14 (12.8)	12 (9.5)	
Yes, former	50 (21.3)	23 (21.1)	27 (21.4)	
ASA classification				
I	19 (8.1)	7 (6.4)	12 (9.5)	.23^c^
II	109 (46.4)	46 (42.2)	63 (50.0)	
III	107 (45.5)	56 (51.4)	51 (40.5)	
Localization tumor				
Larynx	175 (74.2)	75 (68.8)	99 (78.6)	.09^c^
Hypopharynx	61 (25.8)	34 (31.2)	27 (21.4)	
T classification				
T 0	13 (5.5)	10 (9.2)	3 (3.2)	.008^c^
T 1‐2	75 (31.9)	26 23.9)	49 (38.9)	
T 3‐4	147 (62.6)	73 (67.0)	74 (57.9)	
N classification				
N0	152 (64.7)	64 (58.7)	88 (69.8)	.19^c^
N1	17 (7.2)	7 (6.4)	10 (7.9)	
N2	64 (27.2)	37 (33.9)	27 (21.4)	
N3	2 (0.9)	1 (0.9)	1 (0.8)	
AJCC stage^d^				
0	13 (6.0)	10 (9.2)	3 (2.4)	**.009** c
I	28 (11.9)	6 (5.5)	22 (17.5)	
II	35 (14.9)	16 (14.7)	19 (15.1)	
III	35 (14.9)	14 (12.8)	21 (16.7)	
IV	124 (52.8)	63 (57.8)	61 (48.4)	
Indication for TL				
Primary HNC^e^	108 (46.0)	57 (52.3)	51 (40.5)	**.004** c
Recurrent/residual HNC	114 (48.5)	42 (38.5)	72 (57.1)	
Dysfunctional larynx	13 (5.5)	10 (9.2)	3 (2.4)	
Prior treatment				
None	106 (45.1)	55 (50.5)	51 (40.5)	.30^c^
Radiotherapy	106 (45.1)	44 (40.4)	62 (49.2)	
Chemoradiotherapy	23 (9.8)	10 (9.2)	13 (10.3)	
Type of resection				
Laryngectomy (LE)	159 (67.6)	66 (60.6)	93 (73.8)	**.04** c
LE + pharyngectomy	76 (32.4)	43 (39.4)	33 (26.2)	
Closure of neopharynx				
Vertical	129 (54.9)	49 (45.0)	80 (63.5)	.12^c^
T‐closure	21 (8.9)	9 (8.3)	12 (9.5)	
Flap closure	85 (36.2)	51 (46.8)	34 (27.0)	
Lymph node dissection				
None	99 (42.1)	45 (41.3)	54 (42.9)	.46^c^
Unilateral	88 (37.1)	38 (34.9)	50 (39.7)	
Bilateral	48 (20.4)	26 (23.9)	22 (17.5)	
Primary flap reconstruction				**.002** a
No	150 (63.8)	58 (53.2)	92 (73.0)	
Yes	85 (36.2)	51 (46.8)	34 (27.0)	

Continuous variables are represented as mean (SD) and categorical variables are represented as number (percentage of total). Abbreviations: AJCC, American Joint Committee on Cancer; ASA, American Society of Anesthesiologist's physical status classification; BMI, body mass index; SMM, skeletal muscle mass; HNC, head and neck cancer; TL, total laryngectomy.

a Fisher's exact test.

b Independent sample *t* test.

c Pearson Chi squared test.

d Before 2009: according to the 6th AJCC staging manual. After 2009: according to the 7th AJCC staging manual.

e Two patients with primary HNC underwent prior radiotherapy for non‐Hodgkin lymphoma.

**Table 2:** Short‐term and long‐term outcomes after total laryngectomy (TL)

Outcome	All patients (*n* = 235)	Patients with low SMM (*n* = 109)	Patients with normal SMM (*n* = 126)	*P* value
All grade complications	151 (64.3)	74 (67.9)	77 (61.1)	.38^a^
Severe complications^b^	69 (29.4)	38 (34.9)	31 (24.6)	.11^a^
Postoperative mortality	4 (1.7)	4 (3.7)	0 (0)	**.05** a
PCF	64 (27.2)	38 (34.9)	26 (20.6)	**.02** a
Treatment PCF				
Conservative	24 (10.2)	14 (12.8)	10 (7.9)	**.05** c
Surgical	40 (17.0)	24 (22.0)	16 (12.7)	
Duration of hospital stay in days	14 [13‐21]	17 [13‐28]	14 [12‐17]	**<.001** d
Overall survival after TL				
Alive	101 (43.0)	30 (27.5)	71 (56.3)	**<.001** a
Deceased	134 (57.0)	79 (72.5)	55 (43.7)	

Duration of hospital stay was skewed and is represented as median [interquartile range]. Categorical variables are represented as number and percentage of total. Abbreviations: PFC, pharyngocutaneous fistula; SMM, skeletal muscle mass.

a Fisher's exact test.

b Severe complications: Clavien‐Dindo Classification of Surgical Complications grade 3A or higher.

c Pearson Chi squared test.

d Mann‐Whitney *U* test.

### 3.2 Body composition

SMM measurement at the level of C3 was successful in all 235 patients. The lumbar SMI was calculated from the SM area at C3 as described in Section 2. Using the lumbar SMI cutoff point of <43.2 cm^2^/m^2^, 109 patients (46.4% of total) had low SMM. Patients with low SMM had a tendency to be female, had a lower BMI, had a larger primary tumor and AJCC stage, were more likely to have undergone primary TL, and more frequently had flap reconstruction of the pharynx.

### 3.3 Association between low SMM, postoperative complications, PCF, and hospital stay

Table 2 shows the associations between low SMM and outcomes after TL. Patients with low SMM had more severe complications than patients with normal SMM (34.9% vs 24.6%, difference not statistically significant, *P* = .11). All four patients who died in hospital had low SMM (*P* = .05). PCF occurred significantly more often in patients with low SMM than in patients with normal SMM (*P* = .02), and surgical treatment of the PCF was more often necessary (*P* = .05). Hospital stay was significantly longer in patients with low SMM (median, 17 days vs 14 days; *P* < .001).

Logistic regression analysis was performed to identify predictors of the occurrence of PCF. Variables were selected for logistic regression based on known or hypothesized association with PCF. T stage and AJCC stage were not entered in logistic regression analysis because of interaction with the indication for TL. Results of the univariate and multivariate logistic regression analysis are shown in Table 3. In univariate logistic regression analysis, localization of the tumor (hypopharynx), the type of resection (laryngectomy + pharyngectomy), flap closure of the pharynx, low SMM, and a dysfunctional larynx as the indication for TL were significant predictors for PCF. In multivariate logistic regression analysis, only a hypopharyngeal tumor (odds ratio [OR], 3.348; 95% confidence interval [CI], 1.740‐6.443), low SMM (OR, 1.950; 95% CI, 1.038‐3.664), and a dysfunctional larynx (OR, 4.881; 95% CI, 1.375‐17.325) remained significant predictors of PCF.

**Table 3:** Univariate and multivariate logistic regression analysis for the occurrence of pharyngocutaneous fistula (PCF)

Risk factor	Univariate analysis^a^		Multivariate analysis^b^	
	Odds ratio (95% CI)	*P* value	Odds ratio (95% CI)	*P* value
Sex				
Male	1.00 [reference]			
Female	1.633 (0.803‐3.319)	.18	1.091 (4.72‐2.521)	.84
Smoking				
Never	1.00			
Current	1.747 (0.196‐15.583)	.52		
Former	2.048 (0.231‐18.188)	.62		
Alcohol abuse				
Never	1.00			
Yes, current	1.322 (0.534‐3.274)	.55		
Yes, former	1.400 (0.700‐2.802)	.34		
Prior treatment				
None	1.00			
Radiotherapy	1.330 (0.725‐2.473)	.35		
Chemoradiotherapy	1.728 (0.656‐4.550)	.27		
Localization tumor				
Larynx	1.00			
Hypopharynx	3.276 (1.757‐6.107)		3.348 (1.740‐6.443)	**<.001**
Type of resection				
Laryngectomy (LE)	1.00			
LE + pharyngectomy	3.003 (1.646‐5.478)	<.001	1.799 (0.852‐3.798)	.12
Closure of neopharynx				
Vertical	1.00			
T closure	1.492 (0.496‐4.489)	.18	1.581 (0.511‐4.896)	.43
Flap closure	3.238 (1.734‐6.047)	0.001	1.580 (0.525‐4.753)	.42
LND				
None	1.00			
Unilateral	1.458 (0.767‐2.772)	.25		
Bilateral	1.042 (0.469‐2.316)	.92		
Indication for TL				
Primary HNC	1.00			
Recurrent/residual HNC	1.126 (0.614‐2.067)	.70	1.560 (0.809‐3.011)	.19
Dysfunctional larynx	5.046 (1.518‐16.775)	**.008**	4.881 (1.375‐17.325)	**.01**
Low BMI				
<18.5	1.191 (0.373‐3.801)	.77		
18.5‐25.0	0.935 (0.374‐2.340)	.89		
25.0‐30.0	0.479 (0.163‐1.405)	.18		
>30.0	1.00			
Low SMM	2.059 (1.148‐3.692)	**.02**	1.950 (1.038‐3.664)	**.04**

Abbreviations: BMI, body mass index; CI, confidence interval; HNC, head and neck cancer; LND, lymph node dissection; SMM, skeletal muscle mass; TL, total laryngectomy.

a Univariate binary logistic regression analysis.

b Multivariate binary logistic regression (Backward Wald selection model).

### 3.4 Survival analysis

**Figure 2:**
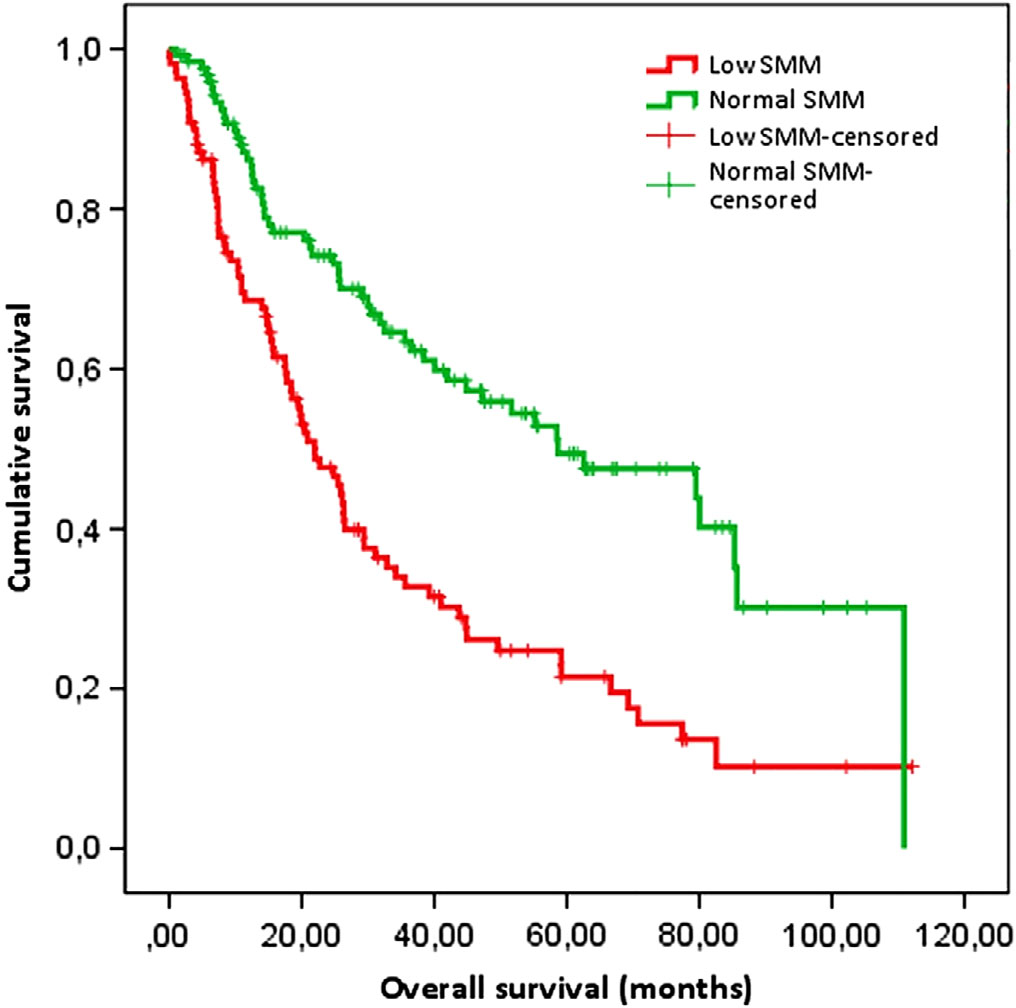
Kaplan‐Meier survival curve for overall survival (OS) after total laryngectomy (TL). Kaplan‐Meier survival curve showing OS in patients with low and normal skeletal muscle mass (SMM). OS for patients with low SMM was the median OS for patients with low SMM after laryngectomy was 18.5 months, compared to 30.1 months in patients with normal skeletal muscle mass (*P* < .001). OS at 5 years after TL was 32.1% for patients with low skeletal muscle mass vs 61.1% for patients with normal skeletal muscle mass (log rank test: *P* < .001) [Color figure can be viewed at wileyonlinelibrary.com]

**Figure 3:**
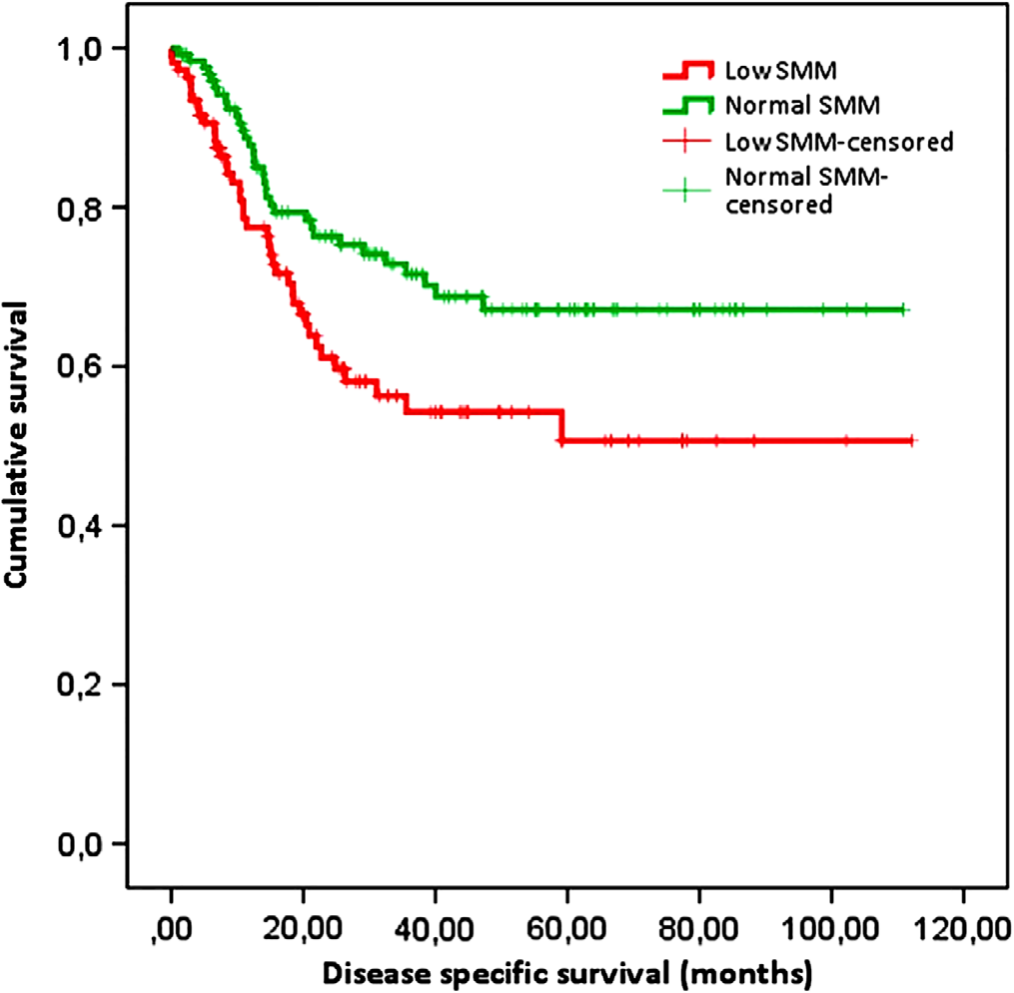
Kaplan‐Meier survival curve for disease‐specific survival (DSS) after total laryngectomy (TL). Kaplan‐Meier survival curves showing DSS in patients with low and normal skeletal muscle mass (SMM). DSS and 5‐year DSS were equal; DSS was 63.3% for patients with low SMM vs 73.8% for patients with normal SMM (log rank test: *P* = .02) [Color figure can be viewed at wileyonlinelibrary.com]

On univariate analysis, OS at the end of follow‐up was significantly lower in patients with low SMM than in patients with normal SMM (median OS was 18.5 months in patients with low SMM vs 30.1 months in patients with normal SMM, *P* < .001), which is visualized in Figure 2. DSS was significantly lower in patients with low SMM, as shown in Figure 3.

Table 4 shows the results of the univariate and multivariate regression analysis for OS. In univariate Cox proportional hazard regression analysis, high N stage (N2 and N3) and low SMM were significant prognosticators for OS. A higher BMI appeared to be associated with longer OS. In multivariate Cox regression analysis, N2 nodal status (OR, 1.494; 95% CI, 1.023‐2.182; *P* = .04), N3 nodal status (OR, 16.040; 95% CI, 3.691‐69.710; *P* < .001) and low SMM (OR, 2.096; 95% CI, 1.494‐2.920; *P* < .001) remained independent prognosticators for decreased OS.

**Table 4:** Univariate and multivariate Cox regression analysis for overall survival

Risk factor	Univariate analysis^a^		Multivariate analysis^b^	
	Odds ratio (95% CI)	*P* value	Odds ratio (95% CI)	*P* value
Sex				
Male	1.00			
Female	0.940 (0.603‐1.465)	.78		
Indication for TL				
Primary HNC	1.00 [reference]			
Recurrent/residual HNC	1.308 (0.923‐1.855)	.13		
Dysfunctional larynx	1.017 (0.439‐2.355)	.97		
N classification^c^				
N0	1.00			
N1	1.771 (0.979‐3.201)	.06	1.452 (0.783‐2.692)	.24
N2	1.763 (1.214‐2.560)	**.004**	1.313 (0.892‐1.932)	**.17**
N3	17.091 (3.939‐74.153)	**<.001**	17.4170 (3.919‐77.410)	**<.001**
Postoperative treatment				
None	1.00			
Radiotherapy	0.756 (0.524‐1.092)	.14		
Chemoradiotherapy	0.834 (0.461‐1.509)	.55		
ASA classification				
I	1.00			
II	1.286 (0.638‐2.590)	.48		
III	1.746 (0.868‐3.511)	.12		
Low BMI				
<18.5	1.237 (0.605‐2.528)	**.56**	0.710 (0.332‐1.568)	.39
18.5‐25.0	1.193 (0.662‐2.150)	**.56**	0.904 (0.487‐1.679)	.75
25.0‐30.0	0.494 (0.246‐0.989)	**.05**	0.538 (0.267‐1.081)	.08
>30.0	1.00			
Low SMM	2.217 (1.566‐3.137)	**<.001**	1.849 (1.202‐2.843)	**.005**

Abbreviations: ASA, American Society of Anesthesiologist's physical status classification; BMI, body mass index; HNC, head and neck cancer; SMM, skeletal muscle mass; TL, total laryngectomy.

a Univariate Cox survival regression analysis.

b Multivariate Cox survival regression analysis (Backward Wald selection model).

c Patients with N3 nodal status: *n* = 2.

## 4 DISCUSSION

It can be anticipated that low SMM has a high prevalence in patients with HNC, at least partly due to the location of the tumor and/or poor physical condition of patients with HNC.^22^, ^23^ Many patients with HNC experience dysphagia and odynophagia, especially after initial (chemo)radiotherapy, leading to malnutrition at diagnosis.^24^, ^25^ A recent study in patients with advanced stage head and neck squamous cell carcinoma undergoing primary chemoradiotherapy, in which SMM was measured at the level of C3, found that 54.5% of patients had low SMM prior to start of treatment.^21^ Another study, in which SMM was measured at the level of L3, found that 77% of patients undergoing TL had low SMM.^26^ In the latter study, low SMM was a significant independent predictor for the occurrence of PCF.^26^ One shortcoming of this study was that abdominal imaging was available in only 57% of patients and consequently only in these patients SMM could be assessed. Because it might be expected that whole body imaging is only routinely performed in selected patients, for example, with advanced stage disease and at high risk of distant metastases, a substantial risk of bias has been introduced.

This study is the largest to date in patients with HNC to show that low SMM is a powerful negative prognostic factor in patients undergoing primary, salvage, or functional TL. Patients with low SMM are at increased risk of developing PCF after TL compared to patients with normal SMM, and the OS of patients with low SMM is only half of the OS of patients with normal SMM. Our data suggest that a simple preoperative measurement of SMM may aid in identifying patients at risk for severe complications, in particular of PCF, and prolonged hospital stay. It may also function as a strong negative prognostic parameter for OS after TL.

Our study is the first to use routinely performed CT or MRI of the head and neck area to assess preoperative muscle status in patients undergoing TL, and the largest to do so in patients with HNC. The main benefit of SMM measurement at the level of C3, compared to SMM measurement at the level of L3, is that almost all patients have appropriate imaging available. In this current study, only 10 out of 245 (4.1%) patients had to be excluded.^26^

The relationship between low SMM and negative short‐term outcomes and prolonged hospital stay is consistent with previous publications in surgical oncology. In abdominal surgery, a recent systematic review and meta‐analysis showed that low SMM is a significant risk factor for major postoperative complications, postoperative mortality, and shorter 1‐year, 3‐year, and 5‐year survival.^15^ In hepatopancreatobiliary cancer, low SMM is associated with reduced OS.^14^ In patients with resectable early stage non‐small‐cell lung cancer, low SMM is associated with poor short‐term and long‐term outcomes after surgery.^27^, ^28^ Low SMM has also been associated with prolonged hospital stay and increased health care‐related costs. A recent study in 452 patients undergoing abdominal surgery for cancer of the alimentary tract showed that patients with low SMM had a significantly longer hospital stay and significantly higher hospital costs than patients with normal SMM.^29^ In our study, patients with low SMM had a significantly longer hospital stay than patients with normal SMM. Although not investigated, it can be anticipated from our results that health care‐related costs have been higher in patients with low SMM as well. Our study also showed a clear relationship with poor OS and DSS in patients with low SMM. This is in line with previous publications in other types of cancer.^30^ This may be due to patients not being fit enough to receive adjuvant treatment, or severe postoperative complications resulting in mortality or delaying necessary adjuvant treatment.

In our study, a cutoff value to define low SMM that has been developed in a separate cohort of Dutch patients with HNC was used.^21^ Although exact definitions and cutoff values for low SMM differ between studies, patient groups and ethnicities, it does appear that low SMM is consistently associated with adverse short‐term and long‐term outcomes in surgical oncology. A potential hypothesis for this may be that patients with low SMM have a decreased capability for recovery after major cancer surgery, for instance due to an altered protein metabolism or a decreased physiological reserve to deal with surgical stress.^31^, ^32^ Another hypothesis is that low SMM indirectly reflects an overall poorer physical functioning in patients, which is not as distinctly found as using other routinely used risk stratification methods.

Future research should be aimed at proactive interventions to improve patient's physical and nutritional status, to clarify whether the adverse effects of low SMM are prognostic only, or if they can be overturned by intensive preoperative optimization and postoperative rehabilitation with physical therapy and nutritional support. Feasibility studies in patients with HNC have shown that resistance training programs in patients undergoing chemoradiotherapy or radiotherapy are feasible and show high patient satisfaction.^33^, ^34^ A randomized controlled trial in patients with lung cancer undergoing short‐term intensive rehabilitation prior to radical surgery showed positive results, with a significant decrease in hospital stay after surgery.^35^ However, most current trials are small; larger randomized controlled trials should clarify whether a multimodal rehabilitation program can reduce the negative effects of low SMM in patients undergoing TL. Alternatively, treatment planning could take the low SMM into account. For example, this could lead the head and neck surgeon to more frequently use overlay pectoralis major flaps for reinforcement in order to decrease the risk of PCF in patients at high risk of PCF.^36^ In our cohort, an overlay pectoralis major flap was only used in nine patients; as such, no conclusions could be drawn of its protective effect.

There are some limitations of our study to discuss. The cutoff value that we used to define low SMM in patients is not a sex‐specific cutoff value. We do believe that a sex‐specific cutoff value would be superior to the cutoff value currently used. However, particularly in female patients, we currently do not have enough data to reliably formulate sex‐specific cutoffs for patients with HNC. We also evaluated using other published sex‐specific and BMI‐specific cutoffs for low SMM, which have been formulated in large cohorts of patients with different types of cancer.^12^, ^37^ Using these cutoffs, approximately 80% of patients are classified as having low SMM. Thus, these cutoffs seem to lose their clinical discriminative power in patients with HNC.

We found some discrepancies between the results of the recent Dutch Head and Neck Society Audit for risk factors of PCF after TL, and our current research.^7^ For instance, previous chemoradiotherapy was a risk factors for PCF occurrence after TL in the Dutch Head and Neck Society Audit, and it was not a significant predictor for PCF in our cohort. This may be explained by the fact that relatively few patients in our cohort had chemoradiotherapy before TL (9.8% in our cohort compared to 15.6% in the Dutch Head and Neck Society cohort). As we did find a slightly higher albeit nonsignificant risk of PCF in patients who had chemoradiotherapy prior to TL, we believe that our analysis was underpowered for this risk factor.

Due to the retrospective nature of the research, all relevant research parameters for body composition or nutritional status may not have been documented or measured during normal clinical practice. In this study, this was particularly true for the plasma albumin level, which was often not measured in our cohort of patients undergoing TL. Also, the ASA classification for physical status was used as a surrogate marker for representing comorbidity. Unfortunately, more specific comorbidity scales could not be determined in this retrospective study because of missing information, particularly in the first 3 years of the study period. Muscle function was not assessed in this study, as muscle function is not tested during routine clinical practice in our institution. Possibly, a measurement of muscle mass and muscle function and/or physical condition (following the geriatric definition of sarcopenia^10^) may provide a more accurate risk profile of muscular status in patients undergoing TL than CT or MRI measured muscle mass alone.

Concerning the imaging techniques used to assess SMM, we decided to include both CT scans and MRI scans of the head and neck area to assess SMM, in order to maximize the number of patients that could be included. Whenever available, we used CT imaging instead of MRI because most research on SMM in patients with cancer is performed using CT imaging. However, the CT measurement method for SMM was formulated on MRI‐based research.^12^, ^13^ Theoretically, there is no difference in SMM between CT imaging and MRI, as both methods are very accurate for SMM assessment. Therefore, we believe it is acceptable to use MRI for SMM measurement when CT imaging is not available. Also, a measurement of SM density, which is the mean HU value of the SM area, was not included in this analysis. A recent study showed that SM area does not differ significantly between CT with and without contrast, but SM density does differ significantly.^38^ As we used both CT with and without contrast for measurement of SMM, we decided not to include SM density in this analysis.

## 5 CONCLUSION

In this study, we found that preoperative low SMM is statistically associated with more frequent PCF, prolonged hospital stay and reduced OS in patients undergoing TL for any indication. A measurement of SMM at the level of C3 allows for routine SMM assessment in diagnostic imaging, without the need for additional abdominal imaging. Our results advocate a preoperative assessment of SMM in patients undergoing TL to identify high risk patients. Possibly, intensive prehabilitation strategies aimed at increasing SMM may reduce these negative outcomes, or wider use of flap reconstruction in patients with low SMM may prevent complications from occurring. Prospective research is needed to evaluate this.

## CONFLICT OF INTEREST

None declared.
